# Sarcinae of the Stomach Contents and Their Diagnostic Value

**Published:** 1915-02

**Authors:** 


**Sarcinae of the Stomach Contents and Their Diagnostic Value.** Graham Chambers, Toronto, Dominion Med. Monthly, January, 1915, concludes as follows:

1. Sarcinae in gastric contents indicate, as a rule, a high degree of stagnation of food in the stomach.
2. The presence of sarcinae in cases characterized by normal or excessive acidity of the gastric juice is, in most cases, due to a benign disease.
3. The presence of sarcinae alone or sarcine along with Boas-Oppler bacilli, in cases of gastric disease of a few months' duration, is frequently due to a malignant process.
4. The finding of both sarcinae and Boas-Oppler bacilli in the gastric contents, characterized by the absence or presence of free hydrochloric acid, may be either due to cancer or peptic ulcer, but is more likely to be the result of the former than the latter disease.

